# The Many Faces of Wnt and Pancreatic Ductal Adenocarcinoma Oncogenesis

**DOI:** 10.3390/cancers3033676

**Published:** 2011-09-21

**Authors:** Colin D. Weekes, Robert A. Winn

**Affiliations:** 1 Division of Medical Oncology, Department of Medicine, University of Colorado Cancer Center, University of Colorado Denver Anschutz Medical Campus, 12801 E. 17^th^ Avenue, Aurora, CO 80045, USA; 2 Division of Pulmonary Sciences and Critical Care Medicine, Department of Medicine, University of Colorado Cancer Center, University of Colorado Denver Anschutz Medical Campus, 12605 E. 16^th^ Avenue, Aurora, CO 80045, USA; E-Mail: robert.winn@ucdenver.edu; 3 Denver Veteran's Affairs Medical Center, 1055 Clermont Street, Denver, CO 80220, USA

**Keywords:** pancreatic ductal adenocarcinoma, Wnt, Frizzled, K-ras, β-catenin, stroma and angiogenesis

## Abstract

Pancreatic ductal adenocarcinoma (PDAC) remains amongst the most lethal human cancers. PDAC is characterized by the tumor mass containing a paucity of malignant cells in association with a large desmoplastic reaction comprised of a variety of stromal components. Sporadic PDAC oncogenesis occurs as a result of the sequential acquisition of genetic aberrations occurring in core genetic pathways. Unfortunately, the average PDAC contains a large number of genetic aberrations that are not uniform between individual cancers. The interplay between the complex genetics and stromal component may represent a significant barrier to the development of effective therapy for this disease and ultimately be an important factor in PDAC lethality. The Wnt pathway has been identified as a one of the common pathways undergoing genetic alterations in PDAC. Wnt is a complex signal transduction pathway utilizing both a β-catenin dependent (canonical) and β-catenin independent (noncanonical) signals to affect a wide array of intracellular events. Wnt signal transduction is an integral component of pancreas organogenesis promoting the expansion and development of the exocrine pancreas. Pancreatic cancer may utilize the Wnt signaling pathway in concert with other signaling pathways such as notch during tumorigenesis. This review will focus on the role of Wnt signal transduction in pancreatic cancer biology.

## 1. Introduction

Pancreatic ductal adenocarcinoma (PDAC) remains amongst the most lethal of human cancers. In 2010 approximately 43,140 individuals were diagnosed with PDAC and 36,800 patients died from the disease [1]. Many of these patients have advanced disease at diagnosis [2]. Unfortunately, despite an increased understanding of pancreatic cancer oncogenesis there has been limited improvement in therapeutic options available to these patients.

## 2. Pancreatic Cancer Oncogenesis

PDAC is distinguishable from most other epithelial tumors in that the tumor mass is comprised predominantly of a large desmoplastic reaction. Approximately 2–10% of PDAC is a result of hereditary syndromes [3,4]. Genetic and histologic analysis of PDAC has defined a sequential set of genetic abnormalities occurring in sporadic PDAC that coincide with the development of preneoplastic lesions known as Pancreatic Intraepithelial Neoplasms (PanIN) [5]. PanIN stage has been demonstrated to correlate with increasing mutational frequency and diversity [6]. Activating mutations in KRAS *(KRAS^G12D^*, *KRAS^G12V^*, *KRAS^G12R^)* occur in upwards of 85% of PDAC and can be recognized in 15–40% of PanIN I lesions [7,8]. PanIN stage 3 lesions are associated with an increased frequency of K-ras mutations as well as the acquisition of p53 and SMAD mutations [5,9]. A genetic mouse model (GEMM), in which the K-ras activating mutation (K-ras*^G12D^*) is placed under the control of pancreas early developmental genes: pancreatic and duodenal homeobox 1 *(Pd×-1)* or p48 (also known as *Ptf1a)*, recapitulates PanIN formation and PDAC observed in humans [10,11]. Interestingly, the addition of p53 mutation to that of K-ras results in the development of sporadic liver metastasis in these mice [12]. Combined these observations demonstrate the sufficiency of K-ras as a central initiator of PDAC oncogenesis in the context of subsequent genetic events that modulate PDAC oncogenesis. Sporadic PDACs harbor a minimum of 60 genetic abnormalities on average involving 12 core genetic pathways [13]. The Wnt pathway represents of these core genetic pathways. Ultimately, the interaction between PDAC complex genetics and the desmoplastic tumor cell microenvironment may be a large determinant of the poor response to currently utilized therapeutic agents to treat this disease.

## 3. Wnt Signal Transduction

The Wnt signal transduction pathway is an important embryonic signaling pathway that is required for proliferation, morphogenesis and differentiation of several organs, including the pancreas [14]. The human Wnt signaling pathway is comprised of 19 Wnt ligands that bind to the frizzled family of receptors and co-receptors [15]. Wnt binding to frizzled results in the activation of two desperate signaling pathways known as the β-catenin dependent (canonical) and the β-catenin independent (non-canonical) pathways. Activation of these intracellular pathways occurs in the context of the co-receptor that forms a complex with frizzled upon Wnt binding. As such, LRP5/6-frizzled complex formation results in β-catenin dependent pathway activation; whereas, formation of the receptor tyrosine kinase-like orphan receptor (ROR) ROR1/2-frizzled complex promotes β-catenin independent pathway activation [16]. Recruitment of disheveled to a juxtaposed position of the β-catenin phosphorylation site promotes β-catenin stabilization by preventing glycogen synthase kinase-3 (GSK-3) mediated phosphorylation of β-catenin. The subsequent inactivation of the cytoplasmic protein complex (comprised of AXIN, adenomatous polyposis coli (APC), GSK-3) that promotes proteosomal degradation of β-catenin allows stabilized unphosphorylated β-catenin accumulate in the cytoplasm. β-catenin can then translocate into the nucleus and bind the transcription factors TCF/LEF resulting in target gene activation [15]. Alternatively, cytoplasmic β-catenin is an integral component of adherent junctions and forms complexes with epithelial-cadherin (E-cadherin) to promote intracellular adhesions [17,18]. In contrast, the β-catenin independent pathway functions to regulate protein kinase C (PKC), calcium mobilization and planar cell polarity [19,20]. The β-catenin dependent pathway has primarily been evaluated in terms of PDAC oncogenesis; however, WNT5a, which classically stimulates the β-catenin independent pathway, is commonly deranged in PDAC supporting the role for β-catenin independent signaling in PDAC tumorigenesis [21,22].

## 4. Wnt Signal Transduction and Pancreatic Cancer Oncogenesis

Mutations in genes encoding for the regulatory proteins of the β-catenin dependent pathway that may function as initiating genetic lesions, as is seen in colon cancer, are rare in PDAC [23]. However, β-catenin accumulation in both the cytoplasmic and nuclear cellular fraction is observed in a small subset of patients and correlates with PanIN grade and the development of PDAC [24-26]. The finding that the combined Wnt and notch signaling pathways are amongst the most common mutated genetic pathways altered in patients with pancreatic cancer suggest it's importance to pancreatic cancer tumorigensis. These pathways appear to be interlinked in that notch signaling functions as a negative regulator β-catenin dependent signaling both in pancreas organogenesis and oncogenesis as will be discussed in a subsequent section. GEMMs have been utilized to interrogate the functional role of Wnt signaling pathway in pancreatic cancer oncogenesis. The findings of these studies have been recently been elegantly reviewed by Morris *et al.* [27]. In short, placing the expression of constitutively active β-catenin with pd×-1 or ptf1 promoter; thereby localizing allelic expression to the pancreas, failed to promote PanIN or malignant tumor formation (Table 1) [28-31]. However, in vivo modeling of pancreatitis in the mouse has led to very unique observations eliciting a novel role for KRAS and Wnt in acinar cell plasticity. In this situation, acinar cells possess the ability to undergo acinar to ductal metaplasia (ADM) [32-34]. This phenomenon allows for the expeditious repair of the pancreasin response to cellular injury, as is observed under conditions of chronic pancreatitis. Chronic pancreatitis has been directly linked to the development of PanINs and PDAC in a KRAS-driven GEMM [35]. Interestingly, enforced expression of high levels of KRAS in acini induces ADM [36]. As such, KRAS mediated ADM may be a crucial component of PDAC development particularly in response to chronic injury or inflammation [35,37,38]. In this setting, β-catenin may serve a similar role as to that in pancreas development. β-catenin is stabilized in response to caerulein induced chronic pancreatitis but is turned off simultaneously to the induction of KRAS mediated ADM. In contrast, forced β-catenin stabilization inhibits ADM, PanIN and PDAC formation, resulting in the development of abnormal ductal structures. These observations further support the inability of β-catenin to initiate pancreas cancer and support the concept that the primary role of β-catenin signaling in PDAC oncogenesis may be in the maintenance and proliferation of PDAC cells.

## 5. WNT5a Signaling and Pancreatic Cancer Biology

WNT5a is a classic β-catenin independent (non-canonical) Wnt that is commonly deranged in pancreatic cancer [21]. Conflicting data exist with respect to its expression in pancreatic cancer. One report demonstrates that Wnt5a expression is downregulated in PDAC cells in comparison to normal tissue [39]. In contrast, other reports demonstrate increased Wnt5a expression in PanIN and PDAC compared to normal counterparts [22]. One explanation in the observe differences in these reports may be a reflection of sampling and number specimens analyzed. Wnt5a may also function in a paracrine function in PDAC. Analysis of PDAC stroma in comparison to the stroma of chronic pancreatitis demonstrates overexpression of Wnt5a in PDAC stroma [40]. Interestingly, coculture experiments with pancreatic cancer cell line and fibroblast suggest that pancreatic cancer cells may induce fibroblast Wnt5a secretion by soluble factors. A potential mechanism may be tumor growth factor-β induced CUTL1 mediated Wnt5a transcription [22]. These observations were associated with the WNT5a regulation of β-catenin dependent function. The characterization of the co-receptors involved in these observations was not performed. However, these data would support recent observations that individual Wnt proteins may function to activate both β-catenin dependent and independent pathways depending on the co-receptor that is complexed with frizzled upon its binding. In this context, WNT5a promotes pancreatic cancer migration, proliferation and invasiveness. The role of β-catenin independent regulation of PDAC progression and metastasis remains unclear.

## 6. Hypoxia, Angiogenesis and Wnt Signaling

The physiologic parameters under which PDAC exist is an important consideration when contemplating the role of a signal transduction pathway in pancreatic adenocarcinoma oncogenesis. How might oxygen tension impact PDAC tumorigenesis in the context of Wnt pathway function? Lesson from pancreas development may apply to PDAC tumorigenesis as both events occur in relative anoxic conditions. Prior to E13.5 pancreas organogenesis occurs under relative hypoxic conditions, which in turn promotes hypoxia inducible factor-1α (HIF-1α) mediated induction of Notch signal transduction [41,42]. Notch in turn, promotes the initial proliferation of undifferentiated endocrine cells from multipotent progenitor cells [43,44]. In contrast, hypoxia results in both HIF-1α dependent and independent inhibition of Wnt/β-catenin signal transduction [45,46]. Beyond day 13.5, neovascularization of the pancreas results in a relative normoxic state which turns off notch signaling in exocrine pancreas cells allowing for the proliferative expansion of the exocrine pancreas in response to Wnt/β-catenin signaling [47,48]. These observations mimic the tight regulation of β-catenin in terms of the temporal relation between its expression and the generation of ADM induced PanIN and PDAC formation. Similarly, HIF-1α is associated with ptf-1a expression and notch pathway activation during acute pancreatitis in mice, which in turn is associated with ADM [49]. The notch pathway regulates epithelial to mesenchymal transition (EMT) as well as promotes the conversion of high-grade PanIN lesions into PDAC in KRAS mutated cells [50,38,51]. Inhibition of both the notch and Wnt/β-catenin signalling pathways can inhibit pancreas cancer cell growth. Thus the interplay between these to embryonic pathways may have a significant role in pancreatic cancer biology. Recently orthotopic models of hypoxic tumor cells from patient-derived xenografts demonstrated increased proliferative capacity and the development of metastases. Selective inhibition of hypoxic cells has been associated with decreased invasiveness and metastatic potential ultimately translating into improved survival in orthotopic mouse models [52,53]. Ultimately, oxygen tension has a significant role in PDAC tumorigenesis by a wide array of mechanisms.

Angiogenesis is an important tumor response to hypoxia. The β-catenin independent pathway may be an integral component of the development functional vasculature as the tumor mass grows. WNT5a has been demonstrated to be responsible for capillary sprouting and branching, which are important components of forming mature vessels during angiogenesis [54]. It may be in this context that WNT5a is overexpressed in pancreas tumor stroma. The restoration of normoxic conditions may directly suppress the aggressive phenotype of PDAC as outlined previously. In addition, treatment of pancreas cancer GEMM with the combination of a hedgehog inhibitor and gemcitabine resulted in tumor vasculature modulation, which in turn enhanced the effectiveness of DNA damaging cytotoxic chemotherapy [55]. Targeting WNT pathway along with hedgehog may be a represent a novel therapeutic strategy to effectively inhibit both pancreas cell tumorigenicity and angiogenesis to further improve the efficacy of gemcitabine-based chemotherapy. This strategy may overcome the apparent clinical inactivity of targeting angiogenesis in PDAC.

## 7. Pancreas Cancer Stem Cells and Chemotherapy Resistance

Pancreatic cancer stem cells (PCSC) have been identified at the tumor-stroma interface within human tumors. The significance of the localization of PCSCs within the pancreas tumor is exemplified by a prevailing hypothesis that stromal components of the tumor can provide appropriate signals to promote tumor cell EMT to promote the development of the stem cell population. These cells are marked by their ability to express cell surface markers CD133, CD24/CD44/ESA or by the functional expression of aldehyde dehydrogenase [56-59]. The PCSC represents a rare cell population of the malignant cells within a tumor mass [60]. The importance of this cell population lies in their ability undergo self-renewal whilst promoting tumor initiation. Self-renewal pathways such as hedgehog, notch and Wnt have been demonstrated to be integral components in the maintenance of cancer stem cell populations. Direct evidence for the function of these pathways in pancreas cancer remains to be fully characterized. However, pharmacologic inhibition of the hedgehog pathway abrogates PCSC tumorigenicity *in vivo* [61]. The interplay between these three pathways may also be very important for the maintenance of this population. Notch has recently been demonstrated to directly bind to β-catenin to inhibit its function by promoting β-catenin lysosomal sequestration and degradation in embryonic stem cells and colon cancer cells [62]. It remains to seen if this finding applies to cancer stem cells but represents an intriguing mechanism by which notch may directly negatively regulate Wnt function. This may be particularly germane to pancreatic cancer given the relationship between notch and Wnt in pancreas organogenesis described previously.

The innate resistance to chemotherapy may be the most clinically relevant function of cancer stem cells. These cells are often quiescent making them refractory to DNA damaging agents. The cancer stem cell population utilizes the ABC drug transporters that pump out chemotherapy. As such, gemcitabine refractory cells possess tumor-initiating properties. These cells also express the chemokine receptor CXCR4, which is an integral component of the ability of these cells to develop metastases in orthotopic models [56]. CXCR4 activation of β-catenin dependent signal transduction results in pancreatic cancer cell proliferation and invasion [63]. Interestingly, CXCR4 mediated signal transduction has been demonstrated to promote gemcitabine resistance in association with β-catenin expression [64]. Oncogenic forms of tyrosine kinase receptors such as c-met, which are expressed on the cellular surface of PCSCs, have been demonstrated to positively regulate β-catenin function [65,66]. Since these same receptors can stimulate survival pathways such as PI3-Kinase/mTOR and the mitogen activated protein kinase (MAPK) pathways, one could envisage that the PCSC niche may function to promote survival of the cancer stem cell during times of cellular stress such chemotherapy exposure. In contrast, the niche may also support EMT and cellular proliferation during favorable microenvironmental conditions to promote PCSC tumorigenicity. Given the role of the Wnt pathway in many of these functions, including the role of the β-catenin independent function in cellular polarization, targeting the Wnt pathway may represent a therapeutic strategy to inhibit pancreas cancer stem cell function at multiple levels.

## 8. Conclusions

In summary, the Wnt pathway is a multifaceted developmental signal transduction pathway controlling a multitude of cellular functions. It is under tight regulation both in pancreas development and pancreatic ductal adenocarcinoma oncogenesis. The exact role of this pathway in PDAC development remains to be fully elucidated and will ultimately be a study in systems biology as it also appears to integrate signals from secondary signal transduction pathways which may invariably contain genetic alterations. In addition, PDAC is comprised of many cellular components all of which play integral but incompletely understood roles in the overall progression of PDAC. Understanding the role of Wnt signaling in the context of these varied biologic events may ultimately result in improved therapeutic strategies for targeting this disease.

## Figures and Tables

**Table 1.** β-catenin Associated Genetic Mouse Models of Pancreas Cancer.

**β-catenin**	**Phenotype**	**Ref.**
Pdx1-Cre*^Late^*; Ctnnb1*^exon3/+^*	Acinar cell proliferation and postnatal pancreatomegaly without tumor formation.	[29]
Pdx1-Cre*Late*; Apc *flox/flox*	Acinar cell proliferation and postnatal pancreatomegaly without tumor formation.	[30]
p48-Cre; Ctnnb1*^exon3/+^*	Development of tumors similar to human solid pseudopapillary tumors.	[31]
p48-Cre;Ctnnb1*^exon3/+^*; LSL-Kras*^G12D^*	Development of tumors similar to intraductal tubular tumors.	[31]
